# Sleep disturbance as a transdiagnostic marker of psychiatric risk in children with neurodevelopmental risk genetic conditions

**DOI:** 10.1038/s41398-022-02296-z

**Published:** 2023-01-11

**Authors:** Samuel J. R. A. Chawner, Alexandra Evans, IMAGINE-ID consortium, Jeanne Wolstencroft, Samuel J. R. A. Chawner, Jeremy Hall, Marianne B. M. van den Bree, Michael J. Owen, David Skuse, F. Lucy Raymond, Nigel Williams, Michael J. Owen, Jeremy Hall, Marianne B. M. van den Bree

**Affiliations:** 1 Centre for Neuropsychiatric Genetics and Genomics, Division of Psychological Medicine and Clinical Neurosciences, Cardiff University, Cardiff, United Kingdom; 2 Neuroscience and Mental Health Innovation Institute, Cardiff University, Cardiff, United Kingdom; 3 NIHR BRC Great Ormond Street Institute of Child Health, University College London, London, UK; 4 School of Clinical Medicine, University of Cambridge, Cambridge, UK; 5 Cambridge University Hospitals NHS Foundation Trust, Cambridge Biomedical Campus, Cambridge, UK; 6 NIHR Bioresource, Cambridge Biomedical Campus, Cambridge, UK

**Keywords:** Psychiatric disorders, Clinical genetics, Predictive markers

## Abstract

Children with rare neurodevelopmental genetic conditions (ND-GCs) are at high risk for a range of neuropsychiatric conditions. Sleep symptomatology may represent a transdiagnostic risk indicator within this patient group. Here we present data from 629 children with ND-GCs, recruited via the United Kingdom’s National Health Service medical genetic clinics. Sibling controls (183) were also invited to take part. Detailed assessments were conducted to characterise the sleep phenotype of children with ND-GCs in comparison to controls. Latent class analysis was conducted to derive subgroups of children with an ND-GC based on sleep symptomatology. Assessment of cognition and psychopathology allowed investigation of whether the sleep phenotypic subgroup was associated with neuropsychiatric outcomes. We found that children with an ND-GC, when compared to control siblings, were at elevated risk of insomnia (ND-GC = 41% vs Controls = 17%, *p* < 0.001) and of experiencing at least one sleep symptom (ND-GC = 66% vs Controls = 39%, *p* < 0.001). On average, insomnia was found to have an early onset (2.8 years) in children with an ND-GC and to impact across multiple contexts. Children in subgroups linked to high sleep symptomatology were also at high risk of psychiatric outcomes (OR ranging from 2.0 to 21.5 depending on psychiatric condition). Our findings demonstrate that children with high genetic vulnerability for neurodevelopmental outcomes exhibit high rates of insomnia and sleep symptomatology. Sleep disruption has wide-ranging impacts on psychosocial function, and indexes those children at greater neuropsychiatric risk. Insomnia was found to onset in early childhood, highlighting the potential for early intervention strategies for psychiatric risk informed by sleep profile.

## Introduction

A number of rare genomic conditions, including recurrent pathogenic copy number variants (CNVs, deletions and duplications >1000 base pairs [1]), and sequence-level variants such as single nucleotide variants (SNVs) have been identified to confer liability for neurodevelopmental and psychiatric conditions including intellectual disability (ID), attention deficit hyperactivity disorder (ADHD), autism and schizophrenia [2–8]. Although individually rare, collectively, these neurodevelopmental risk variants have been implicated in ~15–40% of patients with neurodevelopmental conditions [5, 9]. Although these rare variants are strongly associated with psychiatric conditions, they have incomplete penetrance and exhibit a high degree of pleiotropy, conferring risk for a broad range of psychiatric symptomatology, cognitive deficits, and medical/physical comorbidities across the lifespan [10–15]. Prospective deep phenotyping of children who carry neurodevelopmental risk variants provides a unique opportunity to investigate how psychiatric symptoms emerge during development transdiagnostically, and to identify early endophenotypes. Indeed, sleep functioning has been identified as a potential endophenotype for depression [16], and increased sleep problems have been reported in individuals with autism [17], ADHD [18] and anxiety [19]. There is preliminary evidence from research on 22q11.2 Deletion Syndrome, which confers a high risk for schizophrenia and other neuropsychiatric outcomes, that sleep problems and fatigue are prevalent in carriers [20, 21], and index psychiatric risk [22]. However, it is unknown whether sleep problems are a consistent feature across different neurodevelopmental risk variants.

The relationship between genetic risk, sleep problems and neuropsychiatric outcomes remains to be fully characterised. Here we posit four potential models; (a) the “null model”, whereby sleep problems are not increased in children at genomic risk, (b) the “group risk liability model”, whereby sleep problems are increased in children with a neurodevelopmental risk variant, (c) “individual risk liability model” whereby the presence of sleep problems among children with a neurodevelopmental risk variant predicts individual clinical outcomes, (d)“combined risk liability model” whereby sleep problems are both elevated within children with a neurodevelopmental risk variant and predict individual variability in clinical outcomes.

Here we present findings from a cohort of children with neurodevelopmental risk genetic conditions (ND-GC) from the ECHO (Experiences of CHildren with cOpy number variants) study [13, 23, 24] and the IMAGINE-ID (Intellectual Disability & Mental Health: Assessing the Genomic Impact on Neurodevelopment) study [14, 25, 26], which used identical study methodology and sample assessment [23]. This combined cohort provides an opportunity to apply a genotype-first approach, whereby children were ascertained because of their transdiagnostic genomic risk, and not because of psychiatric diagnosis. Deep phenotyping was conducted, including neurodevelopmental, psychiatric and neurocognitive assessments, thus providing opportunities to study transdiagnostic psychiatric risk. The specific aims of this study were; (1) to characterise the prevalence and nature of sleep problems in children with an ND-GC in comparison to sibling controls; (2) to investigate psychosocial functioning in children with an ND-GC and sleep problems; (3) investigate the relationship between variability in sleep problems and neuropsychiatric outcomes within children with an ND-GC.

### Methodology

The ECHO [13, 23, 24] and IMAGINE-ID [14, 26] studies both recruited families with a child with an ND-GC through all UK National Health Service regional genetics centres. ND-GC was defined as a condition caused by variants which were a) pathogenic or likely pathogenic variants according to the American College of Medical Genetics and Genomics guidelines [27] b) associated with neurodevelopmental outcomes [26]. Previous work from the ECHO and IMAGINE-ID studies has established that this cohort of individuals with an ND-GC are at high risk for a range of psychiatric as well as neurodevelopmental conditions [13, 14, 23, 24, 26]. Families were also recruited via support groups, including Unique, Max Appeal and other groups on social media. Our sample included 629 children with an ND-GC (10.0 years (SD = 3.1, range 6–20 years), 37% female) (see Supplementary Table 1 for full demographics and medical history, including evidence of high rates of neuropsychiatric conditions). Supplementary Table 2 shows the chromosomal variants present in the ND-GC cohort. Variants with *n* ≥ 10 include; 1q21.1 TAR duplication, 1q21.1 distal deletion, 1q21.1 distal duplication, 2p16.3 deletion (NRXN1), 9q34.3 deletion (Kleefstra Syndrome), 15q11.2 deletion, 15q13.3 deletion, 15q13.3 duplication, 16p11.2 proximal deletion, 16p11.2 proximal duplication, 16p11.2 distal deletion, 22q11.2 deletion and 22q11.2 duplication. A sibling without these ND-GCs (sibling control) and closest in age to the index child was also invited to take part. We recruited 183 sibling controls (10.9 years (SD = 2.7, range 6–18 years), 47% female)) (see Supplementary Table 1 for full demographics and medical history). Informed consent was gained from primary carers and participants. The authors assert that all procedures contributing to this work comply with the ethical standards of the relevant national and institutional committees on human experimentation and with the Helsinki Declaration of 1975, as revised in 2008. All procedures involving human subjects/patients were approved by the NHS London Queen Square research ethics committee (14/LO/1069) and South East Wales Research Ethics Committee (09/WSE04/22). ND-GC genotype was confirmed via NHS medical genetics clinic records and by the Cardiff University Division of Psychological Medicine and Clinical Neurosciences (CU DPMCN) laboratory.

### Assessment of sleep, neuropsychiatric and cognitive phenotypes

Assessments of the child were made by experienced research psychologists. Assessments took place within the participant’s home with the advantage that this maximised accessibility to the study and reduced bias against participants who may struggle to travel to a research clinic. A further advantage of home visits was that the child could be assessed in a familiar setting where they were less likely to be anxious and more likely to engage with the assessments. Measures are briefly described here, and full details of all assessments are reported elsewhere [14].

#### Sleep symptomatology

Sleep problems over the preceding three months were established by interviews with the primary carer using the sleep section of the Child and Adolescent Psychiatric Assessment (CAPA) [28]. This section covers insomnias (initial insomnia, trouble initiating sleep; middle insomnia, trouble maintaining sleep; and early insomnia, early morning awakening without being able to return to sleep); hypersomnia (excessive daytime sleepiness, extended sleep duration); restless sleep (inability to get comfortable and feel rested for the night); inadequately rested sleep (lack of restorative and maintained sleep); fatigue and tiredness (feelings of being tired at least half the time, and becoming tired or ‘worn out’ more easily than usual) and parasomnias (nightmares, night terrors and sleepwalking). At the end of this sleep, section, parents were asked whether sleep symptoms significantly impacted their child’s day-to-day functioning, if the response was yes, then further questions were asked about the impact on specific areas of functioning covering (i) the home family environment, (ii) social functioning, (iii) the community, (iv) school, (v) extracurricular sports or clubs, (vi) learning to take care of themselves (vii) play, leisure and recreational activities and (viii) handling of daily chores. For each of these areas of childhood functioning, parents were asked whether sleep symptomatology impacted “*never*”, “*rarely*”, “*sometimes*” or “*often*”, which were assigned scores of 0, 1, 2 and 3, respectively. The CAPA defines insomnia as a sleep disturbance involving a reduction in actual sleep time for a duration of at least 1 h or more, where insomnia had to occur within the last 3 months, and not be the result of an externally imposed change (for example jet lag). For the presence of insomnia, parents were also asked to retrospectively report the age of onset of insomnia and the frequency of occurrence (how many nights in the last 3 months had insomnia occurred).

#### Psychopathology

The CAPA was used to derive categorical diagnoses and a total symptom count composite score, as well as the following symptom subscales: attention deficit hyperactivity disorder (ADHD), anxiety, mood, obsessive-compulsive disorder (OCD) and oppositional defiant disorder (ODD). The child report CAPA was conducted to assess subclinical psychotic experiences. Interviews were taped and diagnoses were confirmed in a consensus meeting led by a child and adolescent psychiatrist. Autism traits were assessed via caregiver reports using the Social Communication Questionnaire (SCQ) [29]. Motor coordination impairment was assessed via caregiver report using the Developmental Coordination Disorder Questionnaire (DCDQ) [30]. The Strengths and Difficulties Questionnaire (SDQ) [31] was completed by the caregiver from which the SDQ total composite score was derived.

#### Cognition

Cognition was assessed via direct child assessments. IQ was assessed using the Wechsler Abbreviated Scale of Intelligence (WASI) [32] from which scores for non-verbal reasoning, perceptual organisation, verbal knowledge and verbal reasoning were derived as well as full-scale IQ (FSIQ), performance IQ (PIQ) and verbal IQ (VIQ) composite scores. Set-shifting ability was assessed using the Wisconsin Card Sorting Test (WSCT) [33]. The CANTAB (Cambridge Neuropsychological Test Automated Battery) [34] was used to assess spatial working memory, spatial planning, sustained attention and reaction time.

### Analysis

#### Sleep phenotype of children with an ND-GC in contrast to controls

The sleep symptomatology profile of children with an ND-GC was established in contrast to controls by examining the prevalence of individual symptoms as ascertained by the CAPA using generalised mixed effect models controlling for sex, age, fixed effects and relatedness (to take into account shared family factors for sibling pairs) as a random effect. For analysis of some sleep symptoms, mixed effect models for binary outcomes failed to converge (often an indicator of low cell counts), and in these instances, we opted for logistic regression models with sex and age as covariates. Furthermore, variables derived from the CAPA concerning sleep symptomatology onset, frequency and impact were also analysed using mixed effect models.

### Latent class analysis in children with an ND-GC

The latent class analysis aimed to group individuals with an ND-GC into categories (classes) based on different patterns of categorical variables. Starting with a single k-class solution, k + 1 solutions were fitted until the optimum solution was reached. Models were run using a robust maximum likelihood parameter estimator and full information maximum likelihood estimation. The optimal number of categories was determined using an adjusted Bayesian information criterion to assess model fit.

### Sleep profiles across different ND-GCs

To investigate the presence of specific genotype-phenotype relationships, analyses were restricted to variant groups with *n* ≥ 10. Chi-squared tests were conducted to examine if sleep subtypes, as derived from the latent class analysis, differed across variant groups. Further analyses were conducted on continuous total sleep symptom count to investigate (a) if all ND-GC groups had elevated symptomatology compared to controls (mixed effect model controlling for sex, age, as fixed effects and relatedness as a random effect) (b) whether ND-GC groups differed in sleep symptomatology and to estimate the proportion of variance ND-GC status explains in total sleep symptom count (ANOVA, sex and age included as covariates).

### Neuropsychiatric and cognitive profiles of sleep subtypes within children with an ND-GC

Categorical psychiatric diagnosis was established using the CAPA, and for autism and developmental coordination disorder, diagnostic cut-offs were applied to the SCQ (≥15 indicative of autism) [35] and DCDQ (age dependent, <8 years: ≤46 indicative of DCD, between 8 and 10 years cut-off = ≤55, ≥10 years ≤57 indicative of DCD [30]). The CAPA (psychiatric traits), SCQ (autism traits), SDQ (childhood psychopathology) and DCDQ (motor coordination difficulties) also provided continuous measures of psychopathology. Cognitive measures (WASI and CANTAB) provided dimensional traits. Logistic regression models, including age and sex as covariates, were conducted to contrast the prevalence of psychiatric conditions between sleep subgroups within children with an ND-GC. For dimensional measures, the scores of ND-GC children were transformed to *z*-scores, standardised for age and sex, and calculated relative to sibling controls. Analysis of variance (ANOVA) models were conducted with the sleep subtype as a predictor and the continuous psychiatric or cognitive trait as the outcome variable, with age and sex as covariates. Post hoc Tukey contrasts with adjustment for multiple testing were conducted to investigate if sleep subtype differed across continuous traits. As a sensitivity analysis, the ANOVA models were repeated in a subgroup (*n* = 479) whereby data was available for maternal education, maternal ethnicity, household income and the presence of sleep medication in the child, to be included as a covariate.

### Multiple testing correction

Benjamini–Hochberg false discovery rate (BH-FDR) multiple testing correction, using an alpha of 0.05, was applied to analyses.

## Results

### Sleep phenotype of children with an ND-GC in contrast to controls

Children with an ND-GC, on average, experienced more sleep symptoms than controls (ND-GC mean = 1.7 symptoms, SD = 1.8), vs control mean = 0.7 symptoms (SD = 1.1), mixed models *p* < 0.001). 66% (415/629) of children with an ND-GC experienced at least one sleep symptom, compared to 39% (72/183) of controls (mixed models *p* < 0.001) (Table 1). 16% (101/628) of children with an ND-GC were receiving medication for sleep problems, compared to 1% (1/183) of controls (*p* < 0.001).

**Table 1:** Sleep symptomatology in children with an ND-GC and control siblings.

Trait	ND-GC	Control	*p* value
	(*N* = 629)	(*N* = 183)	
**Any sleep problem**	66% (415)	39% (72)	**<0.001**
**Insomnia**	41% (255)	17% (31)	**<0.001**
Initial insomnia	25% (156)	15% (28)	**0.018**
Middle insomnia	14% (88)	2% (4)	**<0.001**
Early insomnia	24% (154)	4% (8)	**<0.001**
**Hypersomnia**	8% (53)	2% (4)	0.004*
**Restless sleep**	36% (228)	14% (26)	**<0.001**
**Unrested from sleep**	16% (98)	4% (8)	**<0.001**
**Tiredness**	9% (54)	2% (4)	**<0.001**
**Fatigability**	7% (44)	2% (4)	**0.001**
**Nightmares**	16% (103)	12% (22)	0.302*
**Night Terrors**	9% (55)	3% (5)	**0.001**
**Somnambulism**	8% (50)	9% (16)	0.403

*p* values derived from mixed effect models controlled for sex, age, fixed effects, and relatedness as a random effect. *p* values in bold remained significant following BH-FDR correction for multiple testing.

* mixed effect models for binary outcomes failed to converge, so logistic regression models with sex and age as covariates were conducted.

Children with an ND-GC were more likely than controls to experience elevated rates of insomnia (ND-GC = 41% vs Control = 17%, *p* < 0.001). Insomnia prevalence was elevated at all stages of the sleep cycle; initial insomnia (ND-GC = 25% vs Control = 15%, *p* = 0.018), middle insomnia (ND-GC = 14% vs Control = 2%, *p* < 0.001) and early insomnia (ND-GC = 25% vs Control = 4%, *p* < 0.001). Children with ND-GC also experienced elevated rates of hypersomnia (ND-GC = 8% vs Control = 2%, *p* = 0.004), restless sleep (ND-GC = 36% vs Control = 14%, *p* < 0.001), feelings of being unrested from sleep (ND-GC = 16% vs Control = 4%, *p* < 0.001), feelings of tiredness during the day (ND-GC = 9% vs Control = 2%, *p* < 0.001), fatiguability (ND-GC = 7% vs Control = 2%, *p* < 0.001), and night terrors (ND-GC = 9% vs Control = 3%, *p* = 0.001) compared to controls (Table 1).

For 27% (169/629) of children with an ND-GC, parents reported that sleep symptomatology had a significant impact on the child’s day-to-day functioning, in contrast to 8% (15/183) of controls (mixed models, *p* < 0.001). Parents reported that sleep symptomatology impacted functioning across a range of contexts in both the ND-GC group and controls, in particular, school and home environments (Supplementary Fig. 1). Within individuals where the impact was reported to be significant, the ND-GC group had a higher total impact score, and higher impact scores across the community, self-care, leisure, and household chore contexts (Supplementary Table 3).

Within children who met the criteria for insomnia (ND-GC = 255, Controls = 31), age of onset was reported for 231 children with an ND-GC and 29 sibling controls, and frequency (proportion of days insomnia occurred within a 3-month period) was reported for 238 children with an ND-GC and 30 sibling controls. The age of onset for insomnia was, on average, 2.65 years younger (mixed models, *p* < 0.001) for children with an ND-GC (2.8 years, SD = 3.5) compared to sibling controls (5.5 years, SD = 5.1). The frequency of insomnia within a 3-month period was increased (mixed models *p* = 0.001) in children with ND-GC children (81% of days within a 3-month period) compared to sibling controls (61% of days within a 3-month period).

### Latent class analysis in children with an ND-GC

Latent class growth analysis indicated that a 3-class solution of sleep symptomatology provided the best model fit (adjusted Bayesian information criterion, 5303.259; Vuong-Lo-Mendell-Rubin likelihood ratio test, *p* < 0.001 vs a two-class solution). Supplementary Table 4 displays the symptom profile of each of the three classes derived: *low sleep symptomatology* (Low Sleep Sx, 69% of children with an ND-GC; mean age = 10.0 years (SD = 3.0), 38% female), *high sleep symptomatology, particularly insomnia* (High-Insomnia, 21% of children with an ND-GC; mean age = 9.7 years (SD = 3.2), 32% female), *high sleep symptomatology, particularly tiredness & fatigue* (High-Tiredness, 10% of children with an ND-GC; mean age = 11.3 years (SD = 3.5), 43% female). High-Insomnia and High-Tiredness subgroups overlap in symptom profiles, but High-Insomnia can be distinguished by a higher prevalence of insomnia and parasomnias, whereas High-Tiredness is characterised by a higher prevalence of hypersomnia, tiredness and fatiguability.

### Sleep profiles across different genotypes

**Fig. 1:**
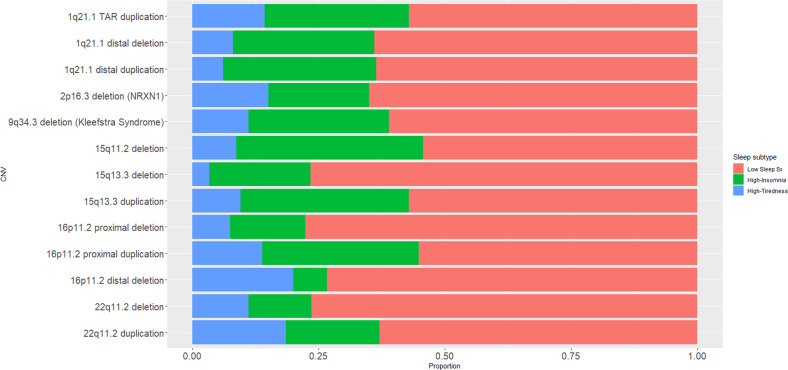
Sleep class profile for each ND-GC genotype. For each ND-GC genotype, the proportion of individuals who have each sleep subtype (Low Sleep Sx, High-Insomnia and High-Tiredness), as derived by latent class analysis.

Supplementary Table 5 and Fig. 1 display for each ND-GC group the proportion of individuals who fall into each sleep class. Sleep class profile was not found to significantly differ by ND-GC (overall *χ*2 = 32.13; *p* = 0.124). However when sleep symptomatology was analysed as a continuous variable, each ND-GC group was found to have elevated total sleep symptom count compared to control siblings (mixed effect model, *p* < 0.05 for all contrasts with control siblings). Furthermore, within children with an ND-GC total sleep symptom count was found to differ by ND-GC group (ANOVA, *p* = 0.014, sex and age included as covariates). However, the proportion of variance in sleep symptom count explained by genotype was low, partial eta-squared = 5%. Post hoc Tukey contrasts did not find any difference in sleep symptom count between reciprocal variants (deletion vs duplication contrasts) at loci where data was available for both reciprocal variants (1q21.1 distal, 15q13.3, 16p11.2 proximal and 22q11.2).

### Neuropsychiatric and cognitive profiles of sleep subtype within children with an ND-GC

Within children with an ND-GC, both the high symptomatology subtypes (High-Insomnia and High-Tiredness) had increased odds of having a neuropsychiatric diagnosis compared to the low sleep symptomatology subgroup (High-Insomnia vs Low Sleep Sx, OR = 3.3, *p* < 0.001; High-Tiredness vs Low Sleep Sx, OR = 3.8, *p* < 0.001). Similarly, there were increased odds for ADHD, anxiety disorder, depression and ODD in both high symptomatology sleep subgroups (see Table 2 for OR and Supplementary Table 6 for diagnosis prevalence within each subgroup), with OR magnitude ranging from 2.0 (High-Tiredness vs Low Sleep Sx, ODD) to 21.5 (High-Tiredness vs Low Sleep Sx, depression). The High-Insomnia subgroup was additionally at increased liklihood for OCD (OR = 2.7, *p* = 0.028), autism (OR = 2.9, *p* < 0.001), and Tic disorder (OR = 2.9, *p* < 0.001) compared to the Low Sleep Sx subgroup. The High-Tiredness subgroup was additionally at increased risk for psychotic experiences (OR = 2.6, *p* = 0.003).

**Table 2:** Odds ratios for neuropsychiatric outcomes for children with an ND-GC with the Low Sleep Sx subgroup as the reference group.

	High-Insomnia vs Low Sleep Sx			High-Tiredness vs Low Sleep Sx		
	OR	95% CI	*p*-value	OR	95% CI	*p*-value
Any Diagnosis^a^	3.3	2.1–5.4	***p*** **<** **0.001**	3.8	2.0–7.8	***p*** **<** **0.001**
ADHD	3.4	2.2–5.2	***p*** **<** **0.001**	3.3	1.9–5.8	***p*** **<** **0.001**
Any Anxiety Disorder	3.1	2.1–4.7	***p*** **<** **0.001**	4.0	2.3–6.9	***p*** **<** **0.001**
Autism^b^	2.9	1.8–4.7	***p*** **<** **0.001**	1.7	0.9–3.0	0.089
DCD^c^	1.9	0.9–4.4	0.120	2.0	0.7–6.9	0.219
Depression	7.3	1.9–35.7	**0.006**	21.5	5.8–103.6	***p*** **<** **0.001**
OCD	2.7	1.1–6.7	**0.028**	0.4	0.0–2.3	0.417
ODD	2.6	1.6–4.1	***p*** **<** **0.001**	2.0	1.0–3.7	**0.034**
Psychotic Experiences	1.6	0.9–2.7	0.113	2.6	1.4–4.8	**0.003**
Tic disorder	2.9	1.7–5.0	***p*** **<** **0.001**	1.2	0.4–2.8	0.721

*p* values derived from logistic regression models taking sex and age into account. *p* values in bold remained significant following BH-FDR correction for multiple testing.

^a^Any diagnosis as derived from the CAPA, does not include autism or DCD, as these are derived from other measures.

^b^As derived from the SCQ.

^c^As derived from the DCDQ.

**Fig. 2:**
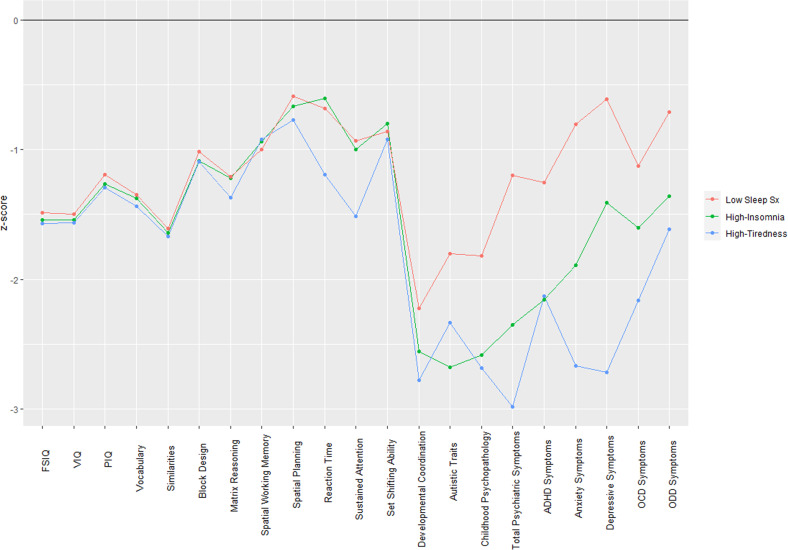
Neuropsychiatric and cognitive profiles for children with a ND-GC presented by sleep class subgroup. For children with a ND-GC, the neuropsychiatric and cognitive profiles of each sleep class subgroup. z-scores were derived relative to the scores of the sibling controls, and were adjusted for age and sex.

Within children with an ND-GC, IQ did not significantly differ across subgroups (*p* = 0.525); Low FSIQ = 80.1 (SD = 14.9); High-Insomnia FSIQ = 79.7 (SD = 13.4); High-Tiredness FSIQ = 77.8 (SD = 13.0). For other continuous traits within children with an ND-GC, the sleep class subgroups did not differ on any of the cognitive traits, but group differences were observed across a range of psychopathology traits; including developmental motor coordination (p = 0.001), autistic traits (*p* < 0.001), childhood psychopathology (SDQ total score, *p* < 0.001), total psychiatric symptoms (CAPA total symptoms, *p* < 0.001), ADHD symptoms (*p* < 0.001), anxiety symptoms (*p* < 0.001), depressive symptoms (*p* < 0.001), OCD symptoms (*p* = 0.009), and ODD symptoms (*p* < 0.001) (See Fig. 2 and Supplementary Table 7). Post hoc contrasts revealed that the majority of these group differences were largely driven by the High-Insomnia and High-Tiredness sleep subgroups having greater neuropsychiatric impairments than the Low Sleep Sx subgroup (Supplementary Table 7). Contrasts between the High-Insomnia and High-Tiredness sleep subgroups found the High-Tiredness subgroup to have greater levels of depressive symptoms (*p* < 0.001), though it should be noted both High-Insomnia and High-Tiredness subgroups had elevated risk for depressive symptoms relative to the Low Sleep Sx subgroup (*p* < 0.001) (see Supplementary Table 7). Results remained largely similar in sensitivity analyses that included additional covariates (See Supplementary Table 8, maternal education, maternal ethnicity, household income and presence of sleep medication in the child).

## Discussion

This study is based on one of the largest research cohorts of children with a rare neurodevelopmental risk chromosomal variant in which sleep symptomatology was assessed, and in which additionally deep phenotyping was conducted across a broad range of psychiatric, neurodevelopmental and cognitive domains. Children were ascertained on the basis of genetic liability rather than psychiatric diagnosis, allowing for prospective transdiagnostic assessment of childhood development and psychiatric outcomes.

This study details the range of sleep symptomatology present across a number of rare neurodevelopmental risk genetic variants. We found that children with an ND-GC had an increased prevalence of insomnia compared to control siblings (41 vs 17%) and were more likely to experience at least one sleep-related symptom compared to control siblings (66 vs 39%). The prevalence of insomnia reported is in line with a recent meta-analysis, whereby 45% of children with a rare genetic condition experienced insomnia [36]. Our study goes a step further, by investigating detailed clinical features of insomnia, and finding that all stages of the sleep cycle are disrupted, with initial (falling to sleep), middle (during the night) and early (morning) insomnia showing increased prevalence in children with an ND-GC compared to controls (initial insomnia 25 vs 15%; middle insomnia 14 vs 2%; early insomnia 24 vs 4%). Furthermore, sleep problems were found to onset early in childhood, particularly for the children with ND-GC in whom the average onset was 2.8 years (compared to 5.5 years for sibling controls). This highlights that many children with insomnia may have longstanding difficulties, but it also points towards the opportunity for early detection of children who are not only at high risk of continuing sleep problems but also psychopathological outcomes. Beyond insomnia, our study details the broad range of sleep symptomatology experienced by children with ND-GC, including increased symptoms of tiredness, fatiguability, restlessness, feeling unrested and night terrors compared to controls. Parents reported sleep problems impacted more on a range of psychosocial contexts for children with ND-GC than their siblings, including in the home, school, community and with peers.

Using sleep symptomatology data, three sleep subtypes could be defined and these corresponded to the transdiagnostic risk of a range of co-occurring psychiatric symptoms. Two high symptomatology subgroups were identified which, although overlapping in symptom profiles, were distinguishable: High-Insomnia was distinguished by a high prevalence of insomnia and parasomnias, whereas High-Tiredness was characterised by a higher prevalence of hypersomnia and extreme feelings of daytime tiredness. Within children with an ND-GC, sleep class subgroup status was found to successfully stratify psychiatric risk. The two high symptomatology subgroups, when contrasted with the low symptomatology subgroup, indexed higher rates of psychiatric conditions (OR = 2.0–21.5), including ADHD (OR = 3.3–3.4), anxiety (OR = 3.1–4.0), depression (OR = 7.3–21.5) and ODD (OR = 2.0–2.6). The two high symptomatology subgroups did not differ from the low symptomatology subgroup in cognitive ability. The High-Insomnia subgroup was additionally at increased liklihood for autism, OCD, and tic disorder, relative to the low sleep symptomatology subgroup (OR = 2.7–2.9). The High-Tiredness subgroup was additionally at increased risk for psychotic experiences (OR = 2.6). Our findings support a “combined risk liability model” whereby sleep problems, (a) are elevated within children with an ND-GC and (b) predict individual variability in psychiatric outcome. This extends previous findings for 22q11.2 Deletion Syndrome, which found that sleep symptoms indexed higher rates of a range of psychiatric conditions [21] and findings of bidirectional relationships between sleep disturbance and psychiatric outcomes in the general population [37–40].

This study has identified subgroups of children with high sleep symptomatology who are also particularly vulnerable to the risk of a transdiagnostic range of psychiatric problems. Within this study design, it is not possible to infer causality, as sleep problems could be an independent marker of psychiatric vulnerability, or a consequence of the onset of psychiatric conditions [37, 41]. However, our findings do indicate that sleep problems often start from a young age, suggesting they may represent an early marker of later atypical psychiatric development. Furthermore, whether or not sleep problems or psychiatric symptoms are the root cause, the presence of sleep problems in the context of psychiatric symptoms requires clinical attention as sleep disturbance is known to exacerbate psychiatric symptoms and lead to worse prognosis in children [42]. By starting with genetic risk, we were able to establish risk indicators based on childhood sleep symptom patterns. This demonstrates the potential of genotype-first study designs for uncovering transdiagnostic risk mechanisms for the development of psychiatric conditions.

Our study finds the profile of sleep symptomatology to be broadly similar across different rare neurodevelopmental risk variants, with genotype accounting for only 5% of the variance. This indicates that sleep problems are not specific to deletions vs duplications. For the loci (1q21.1 distal, 15q13.3, 16p11.2 proximal, and 22q11.2) where data on reciprocal variants were available, sleep problems did not differ between deletion and duplication variants (Fig. 1 and Supplementary Table 5), highlighting a U-shaped relationship between copy number and sleep problems. This is consistent with previous work by our group showing that neurodevelopmental and psychiatric traits are broadly similar across ND-GCs, although subtle differences may be present [14, 43]. Similarly, a meta-analysis of sleep disorders in individuals with rare genetic conditions reported that the rate of insomnia was similar across rare conditions [36]. Our work highlights sleep problems as non-specific features of rare psychiatric risk variants, that often emerge early in development and are associated with markedly increased psychiatric risk.

This work has some limitations. Children with ND-GCs were identified via medical genetic clinics which referral is often for developmental issues, which is likely to introduce ascertainment bias. Furthermore, there is likely to be bias in terms of those families who take part in the research. On the one hand, there could be bias towards higher symptomatology as these children are potentially more likely to be referred for genetic testing, on the other hand, there may be bias towards lower symptomatology as families where the child is experiencing severe mental illness may find it difficult to participate in research. Although our comparison sample of sibling controls to some extent takes into account shared family factors, it does not fully control for potential interaction effects between the rare variant and polygenic variation. The Genes to Mental Health Network is an example of a large-scale international research initiative that will be able to investigate the interaction between rare variants and polygenic risk for psychiatric outcomes [8]. Our reported rates of sleep symptoms may not be representative of the population, but nonetheless, this information has clinical utility for informing families currently receiving genetic diagnoses. Although population-based studies exist [44, 45] they often do not include detailed information on sleep symptoms and rely on medical records. Although the measures we used were gold-standard research-validated psychiatric tools, the assessment of sleep symptoms was based on parent reports which could miss sleep symptomatology reported only by the child [21]. Also, future research should include measures of sensory hypersensitivity, as this could explain associations between neurodevelopmental conditions and sleep disturbance [46, 47]. Furthermore, although we identify sleep as a transdiagnostic marker, the causes of the sleep problems from which our sleep subtypes were derived could have a range of heterogeneous causes. Recent work in children with 22q11.2 Deletion Syndrome found that, during NREM sleep, deletion carriers exhibited increased power in slow delta and sigma oscillations, increased slow-wave and spindle amplitudes, and altered coupling between spindles and slow-waves compared to sibling controls [48], however in this relatively small sample these neurobiological measures of sleep architecture were not associated with clinical measures of sleep symptoms. Future work is needed to (a) better understand the links between clinical sleep symptomatology and neurobiological sleep signatures and (b) expand neurobiological approaches need to be expanded across a range of rare psychiatric variants to determine whether aetiological mechanisms are common across genomic loci.

## Conclusion

This study is one of the largest studies to detail the sleep symptomatology profiles of children with rare neurodevelopmental variants. Our work demonstrates the powerful insights that can be gained from a genotype-first approach, whereby participants are identified based on biological liability irrespective of psychiatric diagnosis and assessed prospectively on a range of domains. Sleep symptomatology, including insomnia, was found to be greatly elevated in children with a high genetic vulnerability. Sleep symptomatology was found to emerge early in childhood development, and impact the child across a range of home, school and community contexts. Sleep symptomatology data successfully stratified children at high genetic vulnerability into subgroups, which in turn indicated a risk for neuropsychiatric diagnosis. This highlights the potential for sleep symptom profiles as a useful tool in personalised medicine approaches for identifying those children at greatest risk of psychiatric conditions and emphasises the important insights that can be gained from studying children at high genomic risk.

## Supplementary information

Supplementary Materials

IMAGINE-ID Consortium

## Data Availability

The full phenotypic IMAGINE dataset is available from the UK Data Archive under special license access (SN 8621) Requests for genotype or linked genotypic-phenotypic data can be made through the study’s data access committee: https://imagine-id.org/healthcare-professionals/datasharing/
