# Supplementary material for: Sleep disturbance as a transdiagnostic marker of psychiatric risk in children with neurodevelopmental risk genetic conditions
Source: Transl Psychiatry. 2023 Jan 11;13:7. doi: 10.1038/s41398-022-02296-z (PMC9834234; doi:10.1038/s41398-022-02296-z)
Supplement: Supplementary file 1 — Supplementary Materials [file 41398_2022_2296_MOESM1_ESM.docx]

**Supplementary Table 1:** Cohort demographics, psychiatric history and medical history

*Any diagnosis as derived from the CAPA, does not include autism as this is derived from other measures

#As derived from the SCQ

$ Categorisation is based on the UK education system and as defined in previous work^1^; Low, O-levels or GCSEs (High School qualifications typically achieved at age 16); Medium, A-levels or Scottish Highers (Qualifications typically achieved at age 18), and vocational training qualification (typically completed following high school); High, university undergraduate and/or postgraduate degree education.

**Supplementary Table 2:** Genotype frequencies

*To preserve anonymity of participants, genotypes with a frequency of less than 5 were included in the other category. The ‘other chromosomal conditions’ category consisted of 46 deletions, 50 duplications with the remaining genetic conditions being caused by either a SNV, translocation, triplication, chromosomal trisomy, or an imprinting disorder. The chromosomal regions affected within the other category were 1p21, 1p36, 1q21, 1q42, 1q44, 2p16, 2p21, 2q11-q21, 2q13, 2q34, 2q37, 3q28-q29, 4p15, 4q28, 5p15, 5q23, 6p25, 6q27, 7p22, 7q11, 8q21, 9p24, 11q14, 12p13, 15pter-q13, 15q11, 15q11-q13, 16p11, 16p12, 16p13, 17p11, 17p13, 17q12, 17q23, 18p11, 22q11, 22q12-q13, 22q13, Xp21, Xp22, Xq28.

**Supplementary Figure 1:** Radar graph of functional impact of sleep symptomatology for children with a ND-GC and sibling controls across a range of contexts


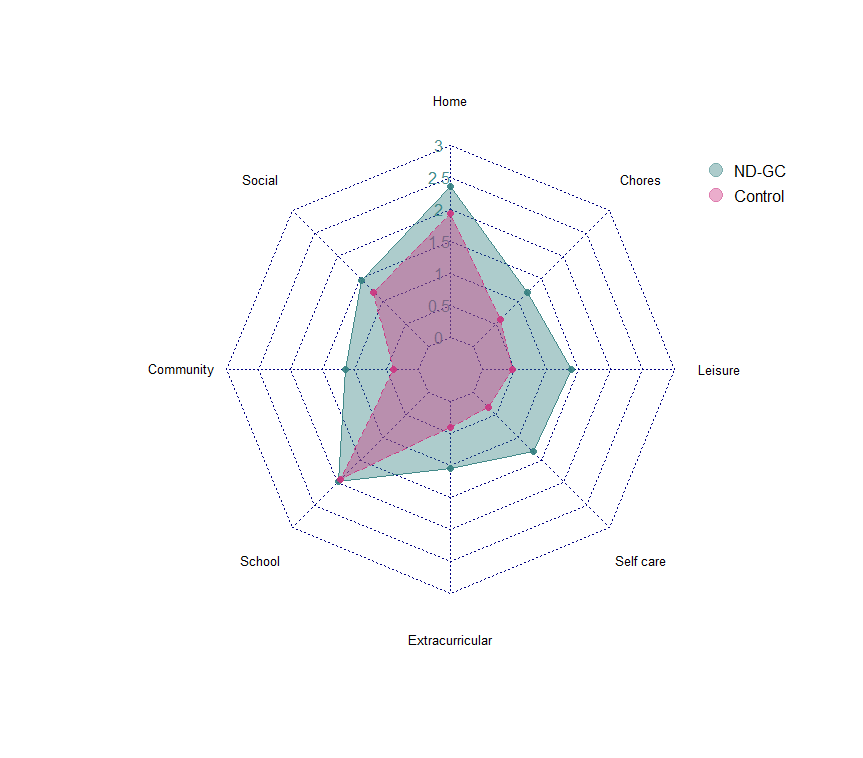


**Supplementary Table 3:** Impact of sleep symptomatology on child functioning across contexts

p-values were derived from mixed models whereby relatedness was controlled for as a random effect, and sex and age were controlled for as fixed effects. p-values in **bold** remained significant following BH-FDR correction for multiple testing.

**Supplementary Table 4:** Sleep symptomatology profiles of the classes derived from latent class analysis

**Supplementary Table 5**: Proportion of individuals in each sleep class within each CNV genotype

**Supplementary Table 6:** Categorical neuropsychiatric diagnoses for children with a ND-GC presented by sleep class subgroup

*Any diagnosis as derived from the CAPA, does not include Autism or DCD as these are derived from other measures

#As derived from the SCQ

^As derived from the DCDQ

**Supplementary Table 7:** Continuous neuropsychiatric and cognitive trait scores for children with a ND-GC presented by sleep class subgroup

p-values derive from Tukey tests applied post hoc to ANOVA models including age and sex as covariates. p-values in **bold** remained significant following BH-FDR correction for multiple testing.

In the last three columns 1 refers to the Low Sleep Sx subgroup, 2 to the High-Insomnia subgroup and 3 to the High-Tiredness subgroup.

#Childhood Psychopathology, as measured by total SDQ score

*****Total Psychiatric symptoms as derived by the CAPA instrument

**Supplementary Table 8:** Sensitivity analysis of phenotypic contrasts between sleep class subgroups taking account of education, maternal ethnicity, household income and presence of sleep medication

p-values derive from Tukey tests applied post hoc to ANOVA models including age, sex, maternal education, maternal ethnicity, household income and presence of sleep medication in the child, as covariates.

values in **bold** remained significant following BH-FDR correction for multiple testing.

In the last three columns 1 refers to the Low Sleep Sx subgroup, 2 to the High-Insomnia subgroup and 3 to the High-Tiredness subgroup.

#Childhood Psychopathology, as measured by total SDQ score

*****Total Psychiatric symptoms as derived by the CAPA instrument

1. Niarchou M, Zammit S, van Goozen SH, Thapar A, Tierling HM, Owen MJ *et al.* Psychopathology and cognition in children with 22q11.2 deletion syndrome. *Br J Psychiatry* 2014; **204**(1)**:** 46-54.
