# Supplementary material for: Sleep disturbance as a transdiagnostic marker of psychiatric risk in children with neurodevelopmental risk genetic conditions
Source: Transl Psychiatry. 2023 Jan 11;13:7. doi: 10.1038/s41398-022-02296-z (PMC9834234; doi:10.1038/s41398-022-02296-z)
Supplement: Supplementary file 2 — IMAGINE-ID Consortium [file 41398_2022_2296_MOESM2_ESM.docx]

**IMAGINE-ID consortium members**

**IMAGINE-ID Consortium author list**

| Surname | Initials | First Name | Title | Institution |
| --- | --- | --- | --- | --- |
| Raymond | F L | F Lucy | Professor | Department of Medical Genetics, University of Cambridge, UK |
| Dewhurst | E | Eleanor | Mrs | Department of Medical Genetics, University of Cambridge, UK |
| Lafont | A | Amy | Ms | Department of Medical Genetics, University of Cambridge, UK |
| Timur | H | Husniye | Ms | Department of Medical Genetics, University of Cambridge, UK |
| Wicks | F | Francesca | Mrs | Department of Medical Genetics, University of Cambridge, UK |
| Ye | Z | Zheng | Dr | Department of Medical Genetics, University of Cambridge, UK |
| Baker | K | Kate | Dr | Department of Medical Genetics, University of Cambridge, UK |
| Walker | N | Neil | Dr | Department of Medical Genetics, University of Cambridge, UK |
| Wallwork | S | Sarah | Ms | Department of Medical Genetics, University of Cambridge, UK |
| Skuse | D | David | Professor | Great Ormond Street Institute of Child Health, University College London, UK |
| Denaxas | S | Spiros | Dr | Institute of Health Informatics, University College London, London, UK |
| Mandy | W | William | Dr | Division of Psychology & Language Sciences, University College London, UK |
| Wolstencroft | J | Jeanne | Dr | Great Ormond Street Institute of Child Health, University College London, UK |
| Davies | S | Sarah | Ms | Great Ormond Street Institute of Child Health, University College London, UK |
| Erwood | M | Marie | Ms | Great Ormond Street Institute of Child Health, University College London, UK |
| Juj | M | Manoj | Mr | Great Ormond Street Institute of Child Health, University College London, UK |
| Kerry | E | Eleanor | Ms | Great Ormond Street Institute of Child Health, University College London, UK |
| Lucock | A | Anna | Ms | Great Ormond Street Institute of Child Health, University College London, UK |
| Printzlau | F | Frida | Ms | Great Ormond Street Institute of Child Health, University College London, UK |
| Srinivasan | R | Ramya | Dr | Great Ormond Street Institute of Child Health, University College London, UK |
| Walker | S | Susan | Dr | Great Ormond Street Institute of Child Health, University College London, UK |
| Coscini | N | Nadia | Dr | Great Ormond Street Institute of Child Health, University College London, UK |
| Fatih | N | Nasrtullah | Mr | Great Ormond Street Institute of Child Health, University College London, UK |
| Denyer | H | Hayley | Ms | Great Ormond Street Institute of Child Health, University College London, UK |
| Andrews | S | Sophie | Ms | Centre for Neuropsychiatric Genetics and Genomics, Division of Psychological Medicine and Clinical Neurosciences, Cardiff University, UK |
| Chawner | SJRA | Samuel | Dr | Centre for Neuropsychiatric Genetics and Genomics, Division of Psychological Medicine and Clinical Neurosciences, Cardiff University, UK |
| Cuthbert | A | Andrew | Dr | Centre for Neuropsychiatric Genetics and Genomics, Division of Psychological Medicine and Clinical Neurosciences, Cardiff University, UK |
| Challenger | A | Aimee | Ms | Centre for Neuropsychiatric Genetics and Genomics, Division of Psychological Medicine and Clinical Neurosciences, Cardiff University, UK |
| Hall | J | Jeremy | Professor | Centre for Neuropsychiatric Genetics and Genomics, Division of Psychological Medicine and Clinical Neurosciences, Cardiff University, UK |
| Lewis | N | Nicola | Ms | Centre for Neuropsychiatric Genetics and Genomics, Division of Psychological Medicine and Clinical Neurosciences, Cardiff University, UK |
| Owen | MJ | Michael | Professor Sir | Centre for Neuropsychiatric Genetics and Genomics, Division of Psychological Medicine and Clinical Neurosciences, Cardiff University, UK |
| Ray | S | Sinead | Ms | Centre for Neuropsychiatric Genetics and Genomics, Division of Psychological Medicine and Clinical Neurosciences, Cardiff University, UK |
| Sopp | M | Matthew | Mr | Centre for Neuropsychiatric Genetics and Genomics, Division of Psychological Medicine and Clinical Neurosciences, Cardiff University, UK |
| Moss | H | Hayley | Ms | Centre for Neuropsychiatric Genetics and Genomics, Division of Psychological Medicine and Clinical Neurosciences, Cardiff University, UK |
| van den Bree | MBM | Marianne | Professor | Centre for Neuropsychiatric Genetics and Genomics, Division of Psychological Medicine and Clinical Neurosciences, Cardiff University, UK |
| Holmans | P | Peter | Professor | Centre for Neuropsychiatric Genetics and Genomics, Division of Psychological Medicine and Clinical Neurosciences, Cardiff University, UK |
| Bowen | S | Samantha | Ms | Centre for Neuropsychiatric Genetics and Genomics, Division of Psychological Medicine and Clinical Neurosciences, Cardiff University, UK |
| Bradley | K | Karen | Mrs | Centre for Neuropsychiatric Genetics and Genomics, Division of Psychological Medicine and Clinical Neurosciences, Cardiff University, UK |
| Birch | B | Philippa | Ms | Centre for Neuropsychiatric Genetics and Genomics, Division of Psychological Medicine and Clinical Neurosciences, Cardiff University, UK |
| Tong | M | Molly | Ms | Centre for Neuropsychiatric Genetics and Genomics, Division of Psychological Medicine and Clinical Neurosciences, Cardiff University, UK |
| Ford | T | Tasmin | Professor | Department of Psychiatry, University of Cambridge |
| Searle | B | Beverly | Dr | Unique Charity, UK |
| Wynn | S | Sarah | Dr | Unique Charity, UK |
| Robertson | L | Lisa | Dr | Aberdeen Royal Infirmary Genetics Service |
| Berg | J | Jonathan | Dr | Ninewells Hospital Dundee Genetics Service |
| Lampe | A | Anne | Professor | Western General Hospital Edinburgh Genetics Service |
| Joss | S | Shelagh | Dr | Glasgow Genetics Centre, Glasgow |
| Brennan | P | Paul | Dr | Northern Genetics Service, Newcastle |
| Kraus | A | Alison | Dr | Yorkshire Regional Genetics Service - Clinical Genetics |
| Weber | A | Astrid | Dr | Cheshire & Merseyside Regional Genetic Service |
| Rawson | M | Myfanwy | Ms | Manchester Centre for Genomic Medicine |
| Quarrell | O | Oliver | Dr | Sheffield Genetic Services |
| Vasudevan | P | Pradeep | Dr | Leicestershire Genetics Centre, Leicester |
| Harrison | R | Rachel | Dr | Nottingham Regional Genetics Service |
| Williams | D | Denise | Dr | West Midlands Regional Genetics Service, Birmingham |
| Maher | E | Eamonn | Professor | East Anglian Medical Genetics Service, Cambridge |
| Kini | U | Usha | Dr | Oxford Genetics Service |
| Clowes | V | Virginia | Dr | London North West Thames Regional Genetics Service |
| Van Dijk | F | Fleur | Dr | London North West Thames Regional Genetics Service |
| Gurasashvilli | J | Jana | Dr | London North East Thames Regional Genetics Service - Great Ormond Street Hospital, London |
| Mansour | S | Sahar | Dr | London South West Thames Regional Genetics Service, St Georges Hospital, Tooting, London |
| Holder-Espinasse | M | Muriel | Dr | London South East Thames Regional Genetics Service Guy's Hospital, London |
| Watford | A | Amy | Dr | Bristol Clinical Genetics Service, Bristol |
| Rankin | J | Julia | Dr | Peninsula Genetics Service, Exeter |
| Baralle | D | Diana | Dr | Wessex Clinical Genetics Service |
| Procter | A | Annie | Dr | All Wales Regional Genetics Service |
